# Damage Evaluation of Concrete under Uniaxial Compression Based on the Stress Dependence of AE Elastic Wave Velocity Combined with DIC Technology

**DOI:** 10.3390/ma14206161

**Published:** 2021-10-17

**Authors:** Guodong Li, Jiarui Gu, Zhengyi Ren, Fengnian Zhao, Yongquan Zhang

**Affiliations:** 1 Transportation Institute, Inner Mongolia University, Inner Mongolia, Hohhot 010070, China; lgd567@imu.edu.cn (G.L.); 31924005@mail.imu.edu.cn (J.G.); justice0224@163.com (Z.R.); imzhaofengnian@outlook.com (F.Z.); 2 Inner Mongolia Engineering Research Center of Testing and Strengthening for Bridges, Inner Mongolia, Hohhot 010070, China

**Keywords:** concrete, damage process, uniaxial compression, AE elastic wave velocity, standard deviation

## Abstract

This study presented evaluation of a concrete damage process by the acoustic emission (AE) technique under uniaxial multi-step compressive loading procedure combined with digital image correlation (DIC). The results showed that AE elastic wave velocity had good stress dependence in the damage process of concrete specimens with different sizes (cube, prism) and coarse aggregate characteristics (volume fraction, maximum size), and the effects of specimen sizes and coarse aggregate characteristics on the stress dependence can be nearly neglected. The standard deviation of 32 AE elastic wave velocities was used as the criterion to evaluate the relative stress ratio of concrete under different damage states, and the damage process of concrete was divided into three damage stages according to this criterion. When the standard deviation is below 70, in the range of 70 to 1700, and greater than 1700, the concrete damage process is defined as steady damage process, accelerated damage process and buckling damage process, respectively. The accuracy of the presented evaluation methodology was demonstrated by comparative results with digital image correlation. The results indicate that the standard deviation of AE elastic wave velocities can potentially serve as a reliable, convenient, and non-destructive evaluation criterion of concrete damage state under uniaxial compressive loading.

## 1. Introduction

The durability, functionality and safety of concrete structures have aging effects, and will deteriorate to different degrees with the duration of service. A large number of engineering practices have shown that with the change in the external environment, and enhancement of the environmental stress of concrete structures beyond a certain stress level, cracks in these rock mass begin to develop, expand, and penetrate, eventually forming intricate weak surfaces and causing serious engineering problems. In order to ensure the long-term stability and economic rationality of these projects, it is necessary to conduct an effective and detailed study on the damage process of concrete structures. To estimate the health status of concrete structures in service, the in-place termly inspection of material properties, residual strength and damage degree is essential [1,2,3]. Non-destructive testing acoustic emission (AE) technology [4,5,6,7] as a non-destructive test method [8,9] is the elastic wave signal generated by analyzing the rapid release of internal strain energy, and is widely used to identify and characterize the damage process of concrete structures or concrete materials [10,11,12]. Therefore, comprehensive testing of the service status, residual strength, and damage location of concrete structures can be realized based on the non-destructive testing technology of AE technology, and this is also very important for fractured concrete structures.

The damage assessment of concrete materials based on acoustic emission technology was currently mostly focused on the time-domain analysis of AE characteristic parameters. The complete damage process of the materials was detected by defining the damage factor and establishing the mathematical relationship with the acoustic emission characteristic parameters. Tetsuya Suzuki [13] established the stress–strain relationship of structural concrete with the Loland model, and the relationship between acoustic emission rate and damage parameters was established based on the rate process analysis. The relative damage of the actual structure was estimated successfully according to the calculated Young’s modulus from the correlation of the data. The damage model established by Weizhen Liu [14] based on cumulative ringing count of acoustic emission was similar to experimentally measured stress-strain curve, which reflected the damage evolution process of coal-fired slag concrete reasonably, and can provide a reference for its theoretical research and engineering applications. The failure mode of the concrete structure can also be judged through the secondary processing and correlation of the AE parameters. Ahmed A. Abouhussien [15] obtained secondary processing data of the amplitude and intensity, B-value analysis, historical index H(t) and severity index Sr to characterize the failure process and damage classification of self-consolidating rubber concrete. Gao Ma [16] performed secondary processing on acoustic emission intensity data, and the cracking process of BFRP-jacketed concrete was characterized by using historical index H(t), severity index Sr and RA-AF. Cluster analysis based on signal characteristics was also used to distinguish the types of damage inside materials. Sena Tayfur [17] revealed the failure mode and damage mechanism of steel fiber concrete by performing cluster analysis on acoustic emission parameters with the k-means algorithm. Arash Behnia [18] determined the peak frequency range of ordinary concrete and steel fiber concrete at each damage stage, and qualitatively tracked the damage process with the fuzzy c-means to cluster the acoustic emission parameters. Furthermore, pattern recognition and signal processing of neural networks based on AE technology have been gradually applied in the destruction analysis of concrete materials and structures. A. Thirumalaiselvi [19] used the SVM algorithm to analyze the AE waveform characteristics, and distinguished the occurrence of new cracks and the expansion of existing cracks by means of the peak frequency, which provided research progress for evaluating the damage location and type of concrete structures. Roman Kravchuk [20] proposed a neural network based acoustic emission event analysis method, which distinguished the damage type and energy dissipation mechanism of fiber-reinforced ultra-high performance concrete successfully. However, the correlation between the acoustic emission stress-elastic wave itself and its excited stress state is rarely studied. It has been proved that the velocity of elastic waves in a medium is highly correlated with the stress state of materials [21,22,23,24]. To study the stress dependence [25,26] of AE elastic wave velocity in concrete is of great significance for obtaining the damage information and can improve the accuracy of concrete’s AE damage position. As the main component of concrete, the properties of coarse aggregate can affect the evolution process of micro-cracks directly, and make the mode of AE elastic wave propagation in concrete more complex [27,28,29].

However, under the condition of compressive stress, it is a long and complicated process for concrete structures to change from initial deformation to final fracture instability. Therefore, it is of engineering significance to predict the stability of concrete structures and to ensure its safety by studying the damage process of concrete materials under compressive stress. Some studies [30,31,32,33] used AE technology to research the damage information of concrete under multi-step axial compressive loading, but they did not provide an effective and reliable method as a reference to evaluate the state of concrete structures. This paper studied the damage process of concrete with different mix proportions under multi-step axial compressive loading by AE technology combined with digital image correlation. The effects of volume fraction of coarse aggregate (i.e., 0%, 20%, 40%, and 60%), maximum size of coarse aggregate (i.e., 9.5 mm, 19 mm, 26.5 mm, and 31.5 mm), and specimen size (i.e., 150 mm × 150 mm × 150 mm, 150 mm × 150 mm × 300 mm) on AE elastic wave velocity were also analyzed. The aim of this paper is to obtain a criterion from the stress dependence of acoustic emission elastic wave velocity in concrete, and use this to evaluate the damage process and damage state of concrete. The achievement of this study provides a new method to evaluate the damage state of the service concrete structure.

## 2. Materials and Methods

### 2.1. Experimental Materials and Mix Proportions

The raw materials used in this study were Portland cement, fly ash, fine aggregate, coarse aggregate, and high-range water reducing agent. The ordinary Portland cement (PO 42.5 cement) and fly ash (Type I) meet Chinese standards. The apparent density, accumulated density, and fineness modulus were 2.65 kg/m^3^, 1630 kg/m^3^, and 2.84, respectively, for fine aggregate. The apparent density, accumulated density, and crushed stone value were 2.76 kg/m^3^, 1850 kg/m^3^, and 17.7%, respectively, for coarse aggregate. Fine aggregate and coarse aggregate were all cleaned by water and then dried. Continuously graded was adopted and coarse aggregate was sieved into six gradations, such as 4.75–9.5 mm, 9.5–16 mm, 16–19 mm, 19–26.5 mm, and 26.5–31.5 mm. The high-range water reducing agent with a water-reducing rate of 25% was used to maintain a consistent slump value of 180 ± 20 mm. As shown in Table 1, eight kinds of concrete mix proportions were designed to explore the influence of coarse aggregate characteristics (volume fraction and maximum size) on stress elastic wave velocity, and six parallel test pieces were set in each mixture. The water–binder ratio of concrete mixture was 0.35. All specimens were de-molded after 24 h, and then placed under standard curing environment (>95% RH, 20 ± 2 °C) until 90 days to be tested.

### 2.2. Test Methods

**Figure 1:**
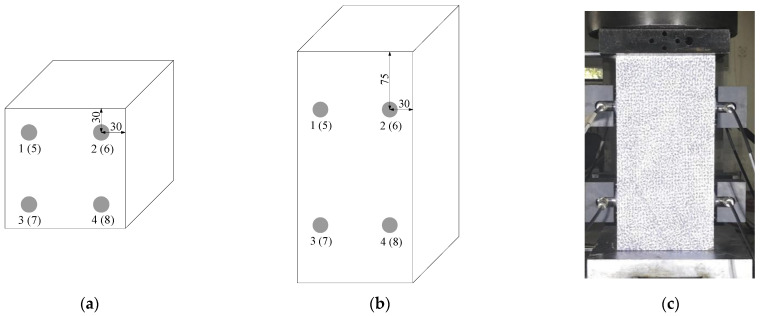
The diagram of experimental layout. (**a**) Cube specimen, (**b**) prism specimen, (**c**) loading diagram.

In this study, the loading system was a double-column bench hydraulic testing machine which is controlled by a force loading method, Micro II Express eight-channel system and DT15I-AST longitudinal wave sensors produced by Physical Acoustics Corporation of United States are used as an AE instrument, the operating frequency is in the range of 100–400 kHz, and the sampling frequency is 5 MSPS, and the detailed experimental parameters are shown in Table 2. The eight sensors were arranged on the two sides of the concrete specimen symmetrically with a spatial positioning layout, and the specific diagram of the experimental layout is shown in Figure 1.

**Figure 2:**
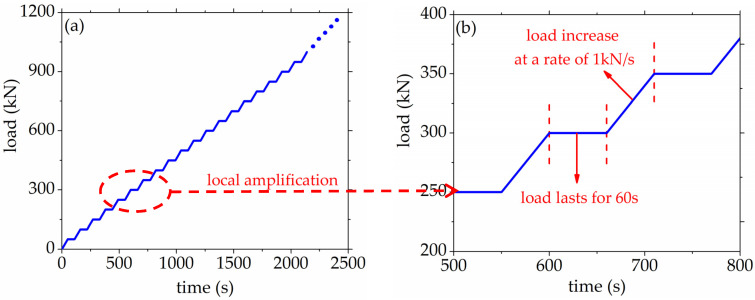
The diagram of the loading scheme. (**a**) load history curve; (**b**) loading gradient details.

In order to obtain the characteristics of AE elastic wave velocity at different stress levels, this experiment designed a stepping load mode. The loading grads was 50 kN, and the loading rate was 1 kN/s. One loading cycle was as follows: the concrete specimen was loaded for 50 s firstly, and then the loading was kept stable for 60 s. The loading cycle was repeated until the specimen was completely damaged. During the voltage stabilization time of the AE testing system, AE elastic wave velocity was collected. The specific diagram of the loading scheme was shown in Figure 2.

**Figure 3:**
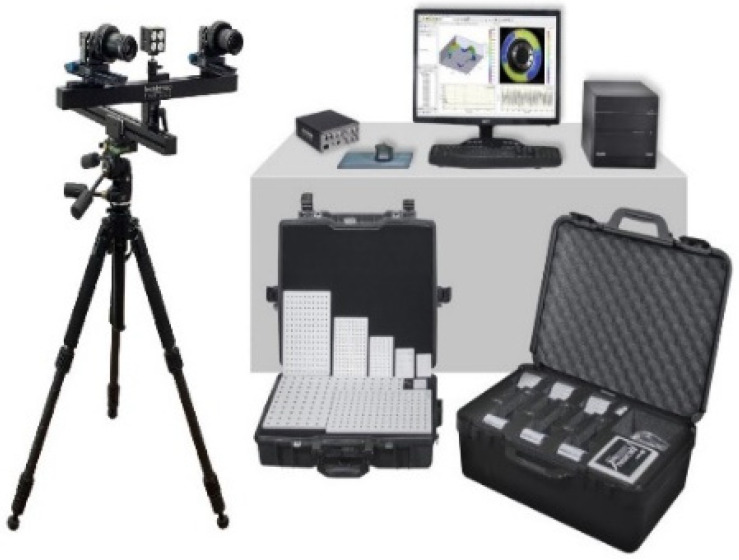
Strain collection system (this picture was provided by the USA CSI company (Jacksonville, FL, USA)).

The surface deformation of the concrete specimen was measured in real time through the digital image correlation method [34,35,36,37,38,39,40] during the loading process, and Figure 3 showed the strain collection system. The instrument is the Vic-3D non-contact full-field strain test system produced by American Correlated Solutions. The speckles were artificially dotted on the surface of the concrete specimen, and the deformation of the speckles were tracked by a high-speed camera which has 5 million pixels. The displacement accuracy is 0.01 pixels, sampling rate is 20 Hz, and the picture quality is 1920 × 2448 pixels.

Displacement and strain were measured by identifying the discrete functions of two digital gray fields. f(x,y) represents the initial image before the object is deformed, which is a discrete function. After being deformed or displaced, it is transformed into another discrete function g(x,y). The point A is one point on the surface of concrete specimen, and the coordinate of this point is set as $f(x_{0},y_{0})$, When the concrete specimen was subjected to compressive loading, the location of the point A changed to that of the point A’ because of the deformation of the concrete specimen surface, and the coordinate of this point was set as $g(x_{0},y_{0})$. The correlation between the two points is represented by the correlation coefficient C. The theoretical relationship between the two discrete functions is expressed by Equation (1), and the calculation formula of the correlation coefficient used as the criterion is shown in Equation (2):

(1) $$g(x^{\prime }\;,\;y^{\prime })-f(x+u(x\;,\;y)\;,\;y+v(x\;,\;y))=0$$

where u(x,y) and v(x,y) represent the displacement field of the figure.

(2) $$C=\frac{\sum f(x,y)\cdot \sum g(x^{\prime },y^{\prime })}{\sqrt{\sum f^{2}(x,y)\sum g^{2}(x^{\prime },y^{\prime })}}$$

where *C* = 1 means correlated completely, and *C* = 0 means irrelevant completely. The surface displacement increment of the concrete specimen is obtained by comparing two images under different loads. The horizontal displacement (*u*) and vertical displacement (*v*) are obtained by calculating the minimum correlation coefficient. 

**Figure 4:**
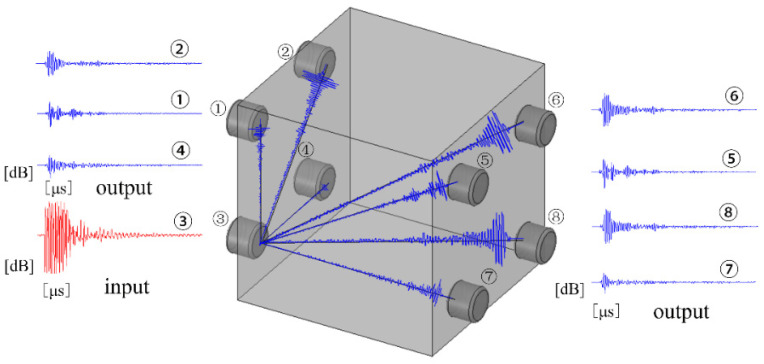
The schematic diagram of AE elastic wave velocity.

The auto sensor test (AST) was carried when the loading was kept stable, and the schematic diagram of the AE elastic wave velocity test is shown in Figure 4. The occurrence and development process of micro-cracks in concrete have stress memory and can directly affect the propagation velocity of the AE elastic wave, this is the theoretical basis of the stress dependence for AE elastic wave velocity. Therefore, the damage process of concrete under axial compression loading can be evaluated by the change of AE elastic wave velocity.

**Figure 5:**
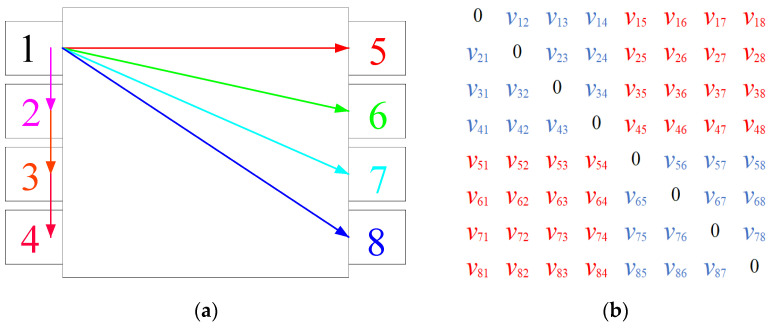
The calculation schematic diagram of AE wave velocity matrix. (**a**) AE signal propagation mode, (**b**) the matrix of AE wave velocity.

One of the transmission paths of elastic wave velocity and the meaning of the overall wave velocity matrix are shown in Figure 5a,b respectively. As shown in Figure 5, the wave velocity obtained by the acoustic matrix test was the overall average wave speed (*ν*_o_), including penetrating wave velocity (*ν*_p_) and surface wave velocity (*ν*_s_). Penetrating wave velocity marked as red symbols is wave velocity between any two sensors located on the opposite surfaces of the concrete specimen, surface wave velocity marked as blue symbols is wave velocity between any two sensors located on the same surfaces of the concrete specimen. However, the mechanism and characteristic of AE wave propagation along the concrete specimen surface is different from that penetrating through the concrete specimen interior. Moreover, the micro-cracks firstly occurred in the concrete specimen interior. Therefore, the penetrating wave velocity more scientifically reflect the propagation characteristics of the stress wave excited by the damage source than the overall average wave velocity. In this study, the average value of AE penetrating wave velocity was defined as the AE elastic wave velocity, and the calculated equation of *ν*_p_ is Equation (3).

(3) $$\nu_{p}=\frac{\left(\sum_{i=1}^{4}\sum_{j=5}^{8}\nu_{ij}+\sum_{m=5}^{8}\sum_{n=1}^{4}\nu_{mn}\right)}{32}$$

The AE elastic wave velocity in this paper is the average value of the penetrating wave velocities from different directions in the concrete.

## 3. Results

### 3.1. Stress Dependency of AE Elastic Wave Velocity

**Figure 6:**
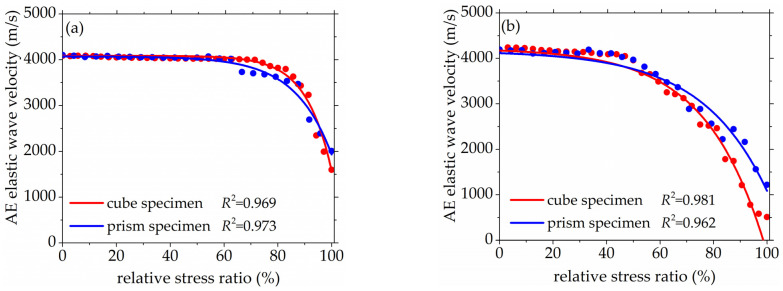

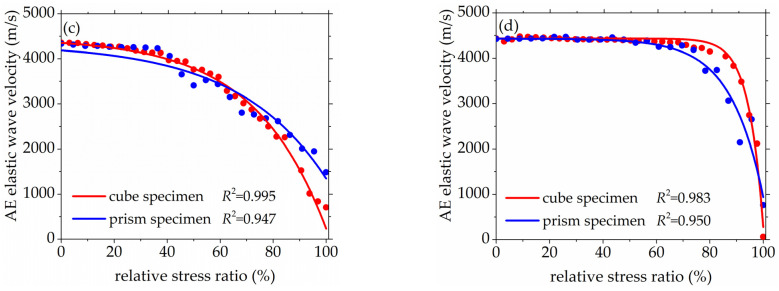
The relationship between AE elastic wave velocity and relative stress ratio with different volume fractions of coarse aggregate, (**a**) 0%, (**b**) 20%, (**c**) 40%, (**d**) 60%.

**Figure 7:**
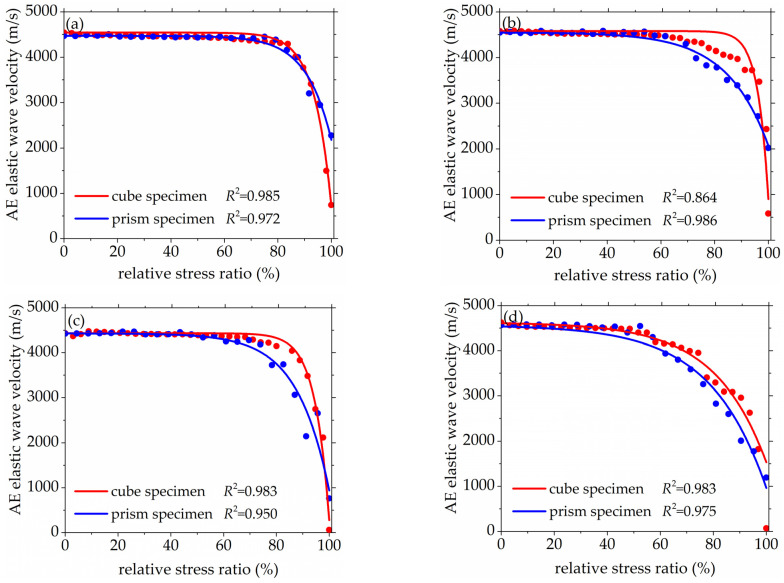
The relationship between AE elastic wave velocity and relative stress ratio with different maximum sizes of coarse aggregate, (**a**) 9.5 mm, (**b**) 19 mm, (**c**) 26.5 mm, (**d**) 31.5 mm.

The AST results of concrete specimens with different mix proportions are shown in Figure 6 and Figure 7, and the experimental results and fitting curves of cube specimens and prism specimens were represented with red line and blue line respectively. In Figure 6, the initial AE elastic wave velocity of the concrete cube specimen with *V*-0%, *V*-20%, *V*-40%, and *V*-60% are 4081 m/s, 4193 m/s, 4359 m/s, and 4436 m/s respectively. The initial AE elastic wave velocity of the concrete prism specimen with *V*-0%, *V*-20%, *V*-40%, and *V*-60% are 4084 m/s, 4195 m/s, 4334 m/s, and 4427 m/s, respectively. It can be found that the AE elastic wave velocity slightly increased with the increase of coarse aggregate volume fraction. In Figure 7, the initial AE elastic wave velocities of the concrete cube specimen with *D*-9.5, *D*-19, *D*-26.5, and *D*-31.5 were 4458 m/s, 4579 m/s, 4626 m/s, and 4651 m/s respectively. The initial AE elastic wave velocity of the concrete cube specimen with *D*-9.5, *D*-19, *D*-26.5, and *D*-31.5 were 4471 m/s, 4547 m/s, 4569 m/s, and 4587 m/s, respectively. It can be found that the AE elastic wave velocity slightly increased with the increasing of coarse aggregate maximum size. This was mainly due to the fact that elastic waves travel faster in the denser medium [41,42]. At the same time, the initial AE wave velocity was an inherent property of concrete with different mix proportions and did not show any size effect because of the layout of the sensors in this study.

In Figure 6 and Figure 7, the AE elastic wave velocities of concrete with different volume fractions and maximum size of coarse aggregate all decreased with the increase of relative stress ratio under axial compression stress. The AE elastic wave velocity–stress curves represent the non-linear tendency obviously, which is mainly due to the formation and development of micro-cracks having a great influence on the propagation of AE elastic waves in concrete. The value of micro-cracks’ size in relation to AE elastic waves’ wavelength determines the effect of micro-cracks on AE elastic waves [43,44,45,46,47]. When the micro-cracks’ size is shorter than the wavelength of AE elastic waves, the appearance of such micro-cracks can inevitably lead to the reduction of concrete elastic properties and AE elastic wave velocity. If micro-cracks’ size is longer than the wavelength of AE elastic waves, the AE elastic wave needs to bypass the micro-cracks and propagates in concrete, but the reflection and the scattering of AE elastic wave will occur around micro-cracks.

According to the fitting results, the relationship between AE elastic wave velocity and relative stress ratio under axial compression stress showed a good exponential function, which is given by:

(4) $$v=v_{p0}+\;ae^{\frac{x}{b}}$$

where $v_{p0}$ is the initial AE elastic wave velocity of concrete under stress-free condition, a and b are the fitting parameters related to the attenuation of AE elastic wave velocity, and *x* represents the stress level, $x=100\times \sigma /\sigma_{\max}$, the correlation coefficients of fitting results are shown in Figure 6 and Figure 7.

Furthermore, in Figure 6 and Figure 7, the AE elastic wave velocity decreased slightly under a lower stress level, the falling range of AE wave velocity started to become bigger when the relative stress ratio become bigger, and the AE elastic wave velocity decreased sharply when the relative stress ratio exceeded a certain value. This phenomenon meant that there were a lot of cracks occurred in concrete with the increasing of compressive strength. At low-stress level, the relative stress ratio is not large enough to cause micro-cracks propagation on the micro-level. Therefore, the AE elastic wave velocity remains nearly stable at this stage. The micro-cracks begin to propagate with the increase of the relative stress ratio. When the micro-cracks in concrete are long enough to exceed the wavelength of the AE elastic wave, the wave velocity decreases obviously. When the relative stress ratio exceeds a certain value, the actual propagation path of AE elastic waves becomes longer as the propagation and connection of micro-cracks, the AE elastic wave velocity decrease sharply.

### 3.2. Damage Process Evaluation of Concrete

The anisotropy of micro-cracks size and distribution can resulted from a certain degree of their preferred orientation under axial compression stress. This phenomenon led to anisotropy and discreteness of AE elastic wave velocity, and formation and development of the micro-cracks were reflected by AE elastic wave velocity. In order to better analyze the damage process of ordinary concrete, this paper uses the standard deviation of AE elastic wave velocity as the damage characteristic value. The standard deviation of AE elastic wave velocity was calculated by Equation (5).

(5) $$\sigma =\sqrt{\frac{1}{N}\sum_{i=1}^{N}{\left(x_{i}-\mu \right)}^{2}}$$

where σ is the standard deviation of the penetrating wave velocity, *N* is the total number of the convective wave velocity (*N* = 32), $x_{i}$ is the specific penetrating wave velocity, and μ is the average value of the penetrating wave velocity in the wave velocity matrix.

**Figure 8:**
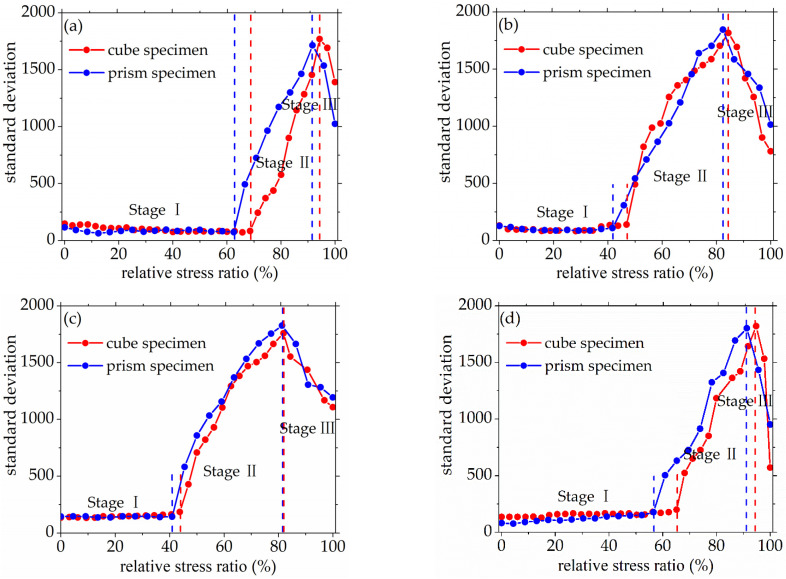
The standard deviations of AE elastic wave velocity for concrete with a different coarse aggregate volume fraction, (**a**) 0%, (**b**) 20%, (**c**) 40%, (**d**) 60%.

**Figure 9:**
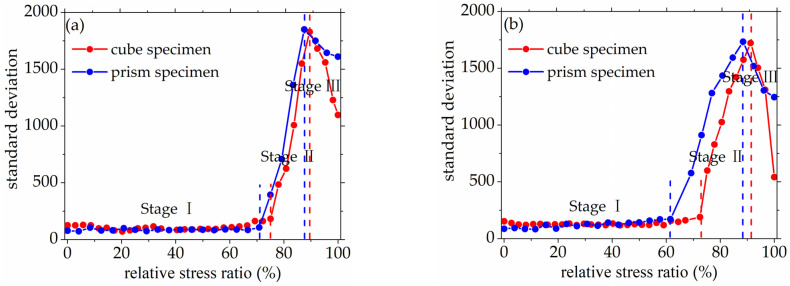

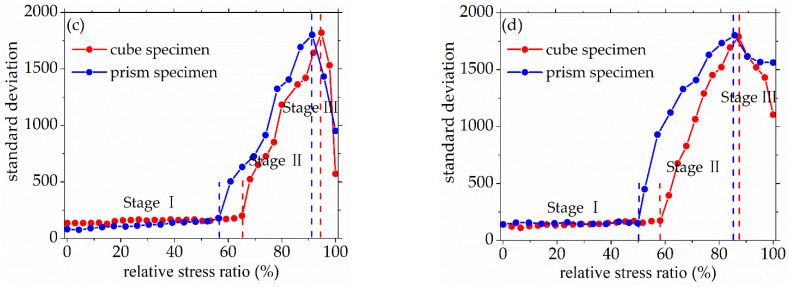
The standard deviations of AE elastic wave velocity for concrete with different coarse aggregate maximum size, (**a**) 9.5 mm, (**b**) 19 mm, (**c**) 26.5 mm, (**d**) 31.5 mm.

As seen in Figure 8 and Figure 9, the standard deviation of AE elastic wave velocity in the damage process of concrete with different mix proportions and specimen sizes showed similar trends. This paper divided the damage process of concrete into three stages, such as steady damage process (stage I), accelerated damage process (stage II) and buckling damage process (stage III). This chapter will analyze in detail the damage process of concrete with different mix proportions and specimen sizes under uniaxial compressive loading.

#### 3.2.1. Steady Damage Process

**Figure 10:**
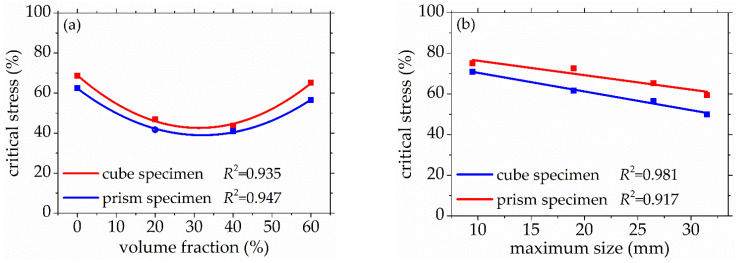
The relative stress ratio of concrete with different mix proportions at the turning point of damage stage I, (**a**) volume fraction, (**b**) maximum size.

**Figure 11:**
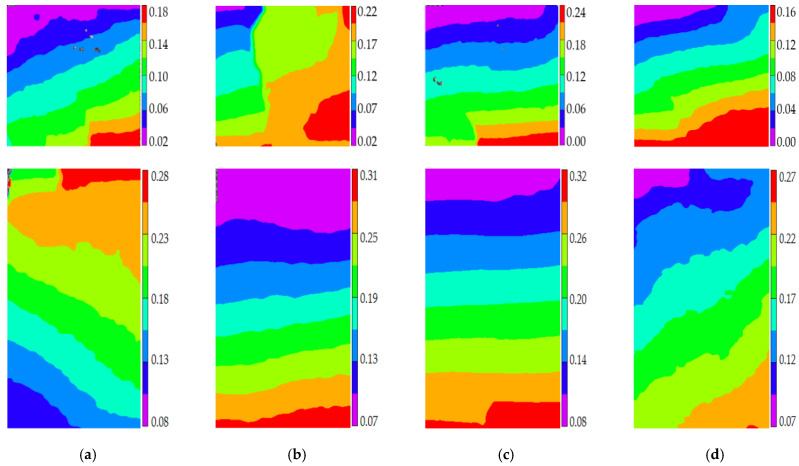
The horizontal strain cloud diagram of concrete with different coarse aggregate volume fraction at the turning point of damage stage I, (**a**) 0%, (**b**) 20%, (**c**) 40%, (**d**) 60%.

**Figure 12:**
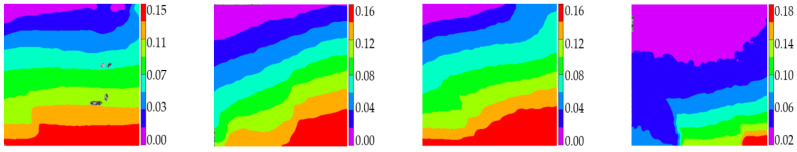

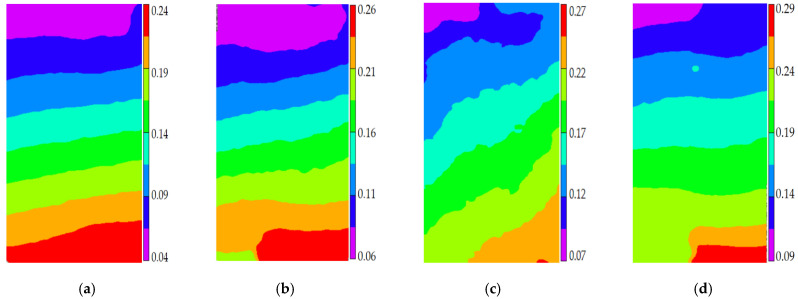
The horizontal strain cloud diagram of concrete with different coarse aggregate volume fraction at the turning point of damage stage I, (**a**) 9.5 mm, (**b**) 19 mm, (**c**) 26.5 mm, (**d**) 31.5 mm.

Stage I is defined as the steady damage process of concrete with different mix proportions and specimen sizes under uniaxial compressive loading in this paper. In this stage, the standard deviation of AE elastic wave velocities changed slightly from Figure 8 and Figure 9. In Figure 10a, the relative stress ratio of the concrete cube specimen with *V*-0%, *V*-20%, *V*-40%, and *V*-60% are 68.5%, 46.8%, 43.7%, and 65.1% respectively. If not otherwise specified in this chapter, these corresponding relative stress ratios are the value of the turning point in the Figure 8 and Figure 9. The relative stress ratio of the concrete prism specimen with *V*-0%, *V*-20%, *V*-40%, and *V*-60% are 62.5%, 41.6%, 40.9%, and 56.5%, respectively. In Figure 10b, the relative stress ratios of the concrete cube specimen with *D*-9.5, *D*-19, *D*-26.5, and *D*-31.5 are 75.0%, 72.4%, 65.1%, and 59.4%, respectively. The relative stress ratio of the concrete prism specimen with *D*-9.5, *D*-19, *D*-26.5, and *D*-31.5 are 70.8%, 61.5%, 56.5%, and 49.8%, respectively. It can be seen that the relative stress ratio is within the scope of 40.9% and 75.0% at the turning point of the damage stage I of concrete with different mix proportions and size. Otherwise, in Figure 11, the maximum horizontal strains of the concrete cube specimen with *V*-0%, *V*-20%, *V*-40%, and *V*-60% are 0.18 mm, 0.22 mm, 0.24 mm, and 0.16 mm, respectively. The maximum horizontal strain of the concrete prism specimen with *V*-0%, *V*-20%, *V*-40%, and *V*-60% are 0.28 mm, 0.31 mm, 0.32 mm, and 0.27 mm, respectively. In Figure 12, the maximum horizontal strain of the concrete cube specimen with *D*-9.5, *D*-19, *D*-26.5, and *D*-31.5 are 0.15 mm, 0.16 mm, 0.16 mm, and 0.18 mm, respectively. The maximum horizontal strain of the concrete prism specimen with *D*-9.5, *D*-19, *D*-26.5, and *D*-31.5 are 0.24 mm, 0.26 mm, 0.27 mm, and 0.29 mm respectively. It can be seen that the maximum horizontal strain is within the scope of 0.15 mm and 0.32 mm at the turning point of the damage stage I of concrete with different mix proportions and size. There are no obvious cracks found in the surfaces of total concrete specimens at the whole damage stage I, even at the turning point.

#### 3.2.2. Accelerated Damage Process

**Figure 13:**
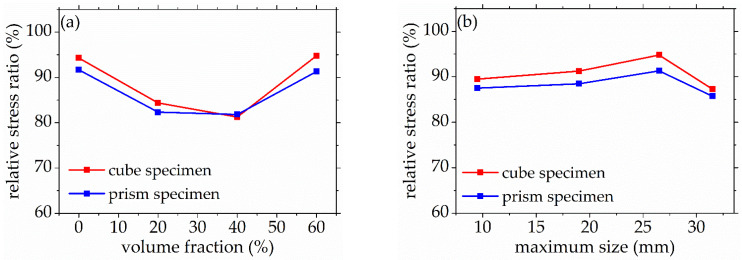
The relative stress ratio of concrete with different mix proportions at the peak point of damage stage II, (**a**) volume fraction, (**b**) maximum size.

**Figure 14:**
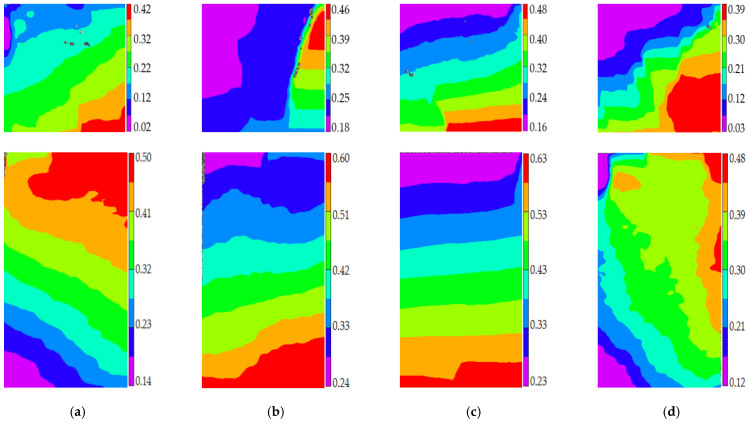
The horizontal strain cloud diagram of concrete with different coarse aggregate volume fraction at the peak point of damage stage II, (**a**) 0%, (**b**) 20%, (**c**) 40%, (**d**) 60%.

**Figure 15:**
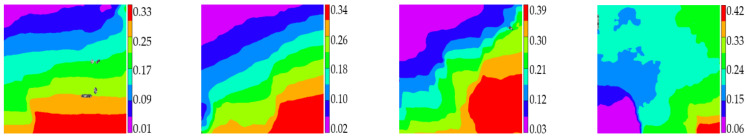

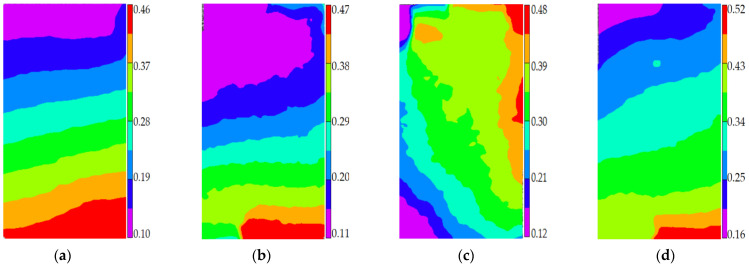
The horizontal strain cloud diagram of concrete with different coarse aggregate volume fraction at the peak point of damage stage II, (**a**) 9.5 mm, (**b**) 19 mm, (**c**) 26.5 mm, (**d**) 31.5 mm.

The stage II is defined as the accelerated damage process of concrete with different mix proportions and specimen sizes under uniaxial compressive loading in this paper. In this stage, the standard deviation of AE elastic wave velocities began to sharply increase until to a maximum value from Figure 8 and Figure 9. In Figure 13a, the relative stress ratios of the concrete cube specimen with *V*-0%, *V*-20%, *V*-40%, and *V*-60% are 94.2%, 84.3%, 81.2%, and 94.7%, respectively. If not otherwise specified in this chapter, these corresponding relative stress ratios are the value of the peak point in Figure 8 and Figure 9. The relative stress ratios of the concrete prism specimen with *V*-0%, *V*-20%, *V*-40%, and *V*-60% are 91.6%, 82.3%, 81.8%, and 91.3%, respectively. In Figure 13b, the relative stress ratios of the concrete cube specimen with *D*-9.5, *D*-19, *D*-26.5, and *D*-31.5 are 89.4%, 91.2%, 94.7%, and 87.2%, respectively. The relative stress ratios of the concrete prism specimen with *D*-9.5, *D*-19, *D*-26.5, and *D*-31.5 are 87.5%, 88.4%, 91.3%, and 85.7%, respectively. It can be seen that the relative stress ratio is within the scope of 81.2% and 94.7% at the peak point of the damage stage I of concrete with different mix proportions and size. Otherwise, in Figure 14, the maximum horizontal strains of the concrete cube specimen with *V*-0%, *V*-20%, *V*-40%, and *V*-60% are 0.42 mm, 0.46 mm, 0.48 mm, and 0.39 mm, respectively. The maximum horizontal strains of the concrete prism specimen with *V*-0%, *V*-20%, *V*-40%, and *V*-60% are 0.50 mm, 0.60 mm, 0.63 mm, and 0.48 mm, respectively. In Figure 15, the maximum horizontal strains of the concrete cube specimen with *D*-9.5, *D*-19, *D*-26.5, and *D*-31.5 are 0.33 mm, 0.34 mm, 0.39 mm, and 0.42 mm, respectively. The maximum horizontal strains of the concrete prism specimen with *D*-9.5, *D*-19, *D*-26.5, and *D*-31.5 are 0.46 mm, 0.47 mm, 0.48 mm, and 0.52 mm, respectively. It can be seen that the maximum horizontal strain is within the scope of 0.33 mm and 0.63 mm at the peak point of the damage stage II of concrete with different mix proportions and size. The micro-cracks began to occur in the surface of concrete specimens at the damage stage II, and were obviously found in the surfaces of the total concrete specimens at the peak point.

#### 3.2.3. Buckling Damage Process

**Figure 16:**
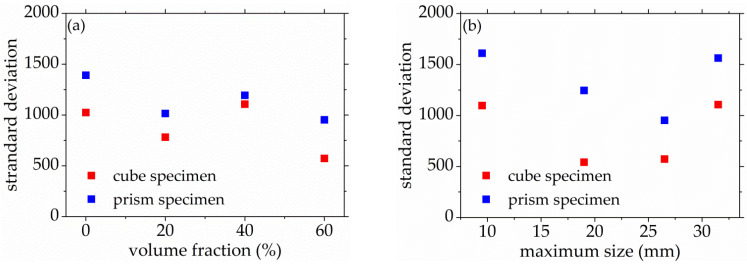
The standard deviation of AE elastic wave velocity of concrete with different mix proportions at the final failure state, (**a**) volume fraction, (**b**) maximum size.

**Figure 17:**
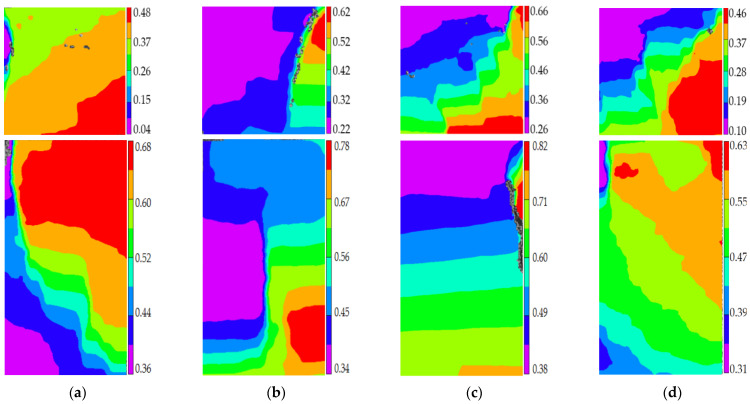
The horizontal strain cloud diagram of concrete with different coarse aggregate volume fraction at the completely failure state, (**a**) 0%, (**b**) 20%, (**c**) 40%, (**d**) 60%.

**Figure 18:**
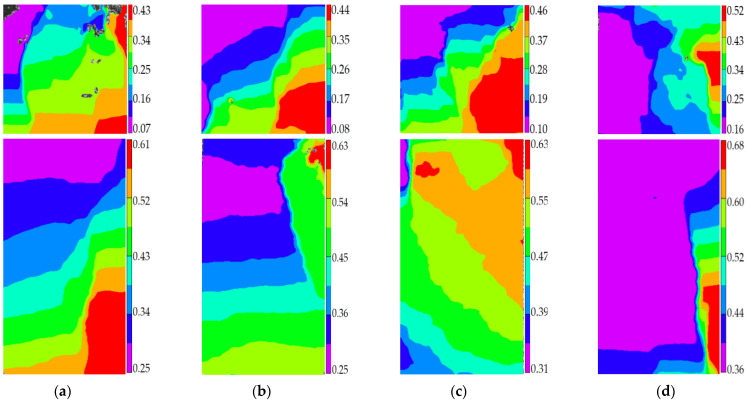
The horizontal strain cloud diagram of concrete with different coarse aggregate volume fraction at the completely failure state, (**a**) 9.5 mm, (**b**) 19 mm, (**c**) 26.5 mm, (**d**) 31.5 mm.

The stage III is defined as the buckling damage process of concrete with different mix proportions and specimen sizes under uniaxial compressive loading in this paper. In this stage, the standard deviation of AE elastic wave velocities began to sharply decrease from Figure 8 and Figure 9, and the width of concrete surface cracks gradually propagated until they were completely damaged. In Figure 16, the standard deviation of AE elastic wave velocities of cube specimens and prism specimens nearly all decreased with the increase of volume fraction and maximum size of coarse aggregate, and the standard deviations of AE elastic wave velocities of some specimens increased. This phenomenon can be attributed to the interfacial transition zone, specimen size effect and crack characteristic. From the Figure 17 and Figure 18, the horizontal strain was much larger than the value of Figure 11, Figure 12, Figure 14 and Figure 15. In Figure 17, The largest horizontal strain of cube specimens reached 0.66 mm, and the largest horizontal strain of prism specimens reached 0.82 mm. In Figure 18, the largest horizontal strain of cube specimens and prism specimens reached 0.52 mm and 0.68 mm, respectively.

### 3.3. The Threshold Value of Concrete Damage

**Figure 19:**
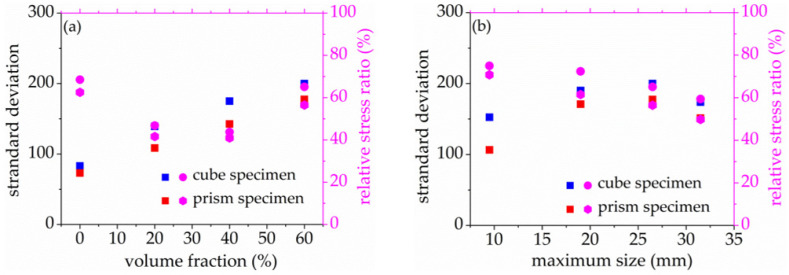
Standard deviation of acoustic emission penetrating wave velocity of concrete with different mix proportion at the turning point, (**a**) volume fraction, (**b**) maximum size.

In Figure 19a, the standard deviation of AE elastic wave velocity of the concrete cube specimens with *V*-0%, *V*-20%, *V*-40%, and *V*-60% are 83, 139, 175, and 200, respectively. The standard deviation of AE elastic wave velocity of the concrete prism specimen, with *V*-0%, *V*-20%, *V*-40%, and *V*-60% are 73, 108, 142, and 177, respectively. It can be found that the AE elastic wave velocity slightly increased with the increase of coarse aggregate volume fraction. In Figure 19b, the standard deviation of AE elastic wave velocity of the concrete cube specimen with *D*-9.5, *D*-19, *D*-26.5, and *D*-31.5 are 152, 190, 200, and 174 respectively. The standard deviation of AE elastic wave velocities of the concrete prism specimen with *D*-9.5, *D*-19, *D*-26.5, and *D*-31.5 are 106, 171, 177, and 152, respectively. It can be seen that the standard deviation of AE elastic velocity is within the scope of 73 and 200 at the critical point of the damage stage I of concrete with different mix proportions and size. This paper defined 70 as the waring threshold value of stage I transfer to stage II.

**Figure 20:**
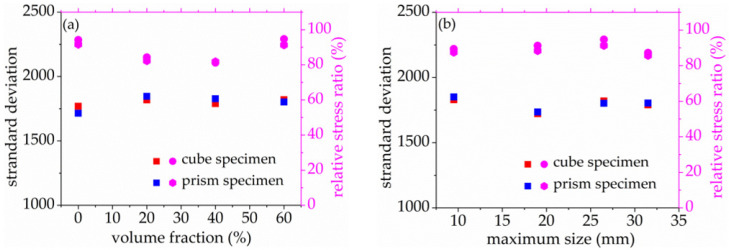
Standard deviation of acoustic emission penetrating wave velocity of concrete with different mix proportion at peak point, (**a**) volume fraction, (**b**) maximum size.

In Figure 20a, the standard deviation of AE elastic wave velocity of the concrete cube specimen with *V*-0%, *V*-20%, *V*-40%, and *V*-60% is 1769, 1817, 1789, and 1819, respectively. The standard deviation of AE elastic wave velocity of the concrete prism specimen with *V*-0%, *V*-20%, *V*-40%, and *V*-60% is 1714, 1846, 1827, and 1802 respectively. It can be found that the AE elastic wave velocity slightly increased with the increase of coarse aggregate volume fraction. In Figure 20b, the initial AE elastic wave velocities of the concrete cube specimen with *D*-9.5, *D*-19, *D*-26.5, and *D*-31.5 are 1829, 1722, 1819, and 1790, respectively. The initial AE elastic wave velocity of the concrete cube specimen with *D*-9.5, *D*-19, *D*-26.5, and *D*-31.5 are 1850, 1734, 1801, and 1803, respectively. It can be seen that the standard deviation of AE elastic velocity is within the scope of 1714 and 1850 at the critical point of the damage stage II of concrete with different mix proportions and size. This paper defined 1700 as the waring threshold value of stage II transfer to stage III.

When the standard deviation of AE elastic wave velocity is in the scope of 73 and 200, the relative stress ration of concrete with different coarse aggregate characteristic and specimen size was in the scope of 40.5% and 75% at the turning point of stage I. When the standard deviation of AE elastic wave velocity was in the scope of 1714 and 1850, the relative stress ration of concrete with different coarse aggregate characteristic and specimen size was in the scope of 81.2% and 94.7% at the peak point of stage II. Therefore, this study also defined the standard deviation 70 and 1700 as the warning threshold of concrete at about 40% and 81% stress level, respectively. This definition can have a good promotion effect to the evaluation of concrete materials.

## 4. Discussions

In this paper, the damage process of concrete under graded axial compression loading was analyzed based on the stress dependence of AE elastic wave velocity. The effects of specimen size and coarse aggregate characteristics were considered, and the conclusions can be drawn as follows:

(1) The AE elastic wave velocity in concrete with different mix proportion and specimen size is stress-dependent, and the relationship between AE elastic wave velocity and relative stress ratio showed a good exponential function. The effects of coarse aggregate characteristic and specimen size on the stress dependence of AE elastic wave velocity can be nearly neglected.
(2) In this study, the standard deviation of 32 AE elastic wave velocities was defined as the evaluation criterion of the concrete damage process, and the whole damage process was divided into three stages by this evaluation criterion, such as a steady damage process, accelerated damage process and buckling damage process. The two key values of the standard deviation were 70 and 1700, When the standard deviation is below 70, in the range of 70 to 1700, and greater than 1700, the concrete damage process is defined as a steady damage process, accelerated damage process and buckling damage process respectively.
(3) The changing history of horizontal strain verified the scientificity and reliability of this evaluation criterion. The relationship between standard deviation and relative stress ratio of the turning point and the peak point of the concrete damage process were analyzed, and the results showed that the standard deviation of AE elastic wave velocity can also be used to evaluate the stress ratio when concrete was under axial compressive loading.

## Figures and Tables

**Table 1:** The mix proportions of concrete.

Mixture Code	Cement (kg/m^3^)	Water (kg/m^3^)	Sand (kg/m^3^)	Fly Ash	Volume Fraction (%)	Maximum Size (mm)	Compressive Strength (MPa)	
							Cube	Prism
*V*-0%	716	300	1289	143	0	—	74.1	68.8
*V*-20%	631	265	1137	126	20	26.5	65.8	62.3
*V*-40%	547	229	985	109	40		61.7	59.7
*V*-60%	463	194	833	92	60		76.3	71.8
*D*-9.5	463	194	833	92	60	9.5	65.1	62.4
*D*-19						19	71.7	65.7
*D*-26.5						26.5	76.3	71.8
*D*-31.5						31.5	66.3	62.2

**Table 2:** The setting value of acoustic emission (AE) parameters.

Parameter	Threshold (dB)	Amplification (dB)	Floating Threshold (dB)	PDT (μs)	HDT (μs)	HLT (ms)
Value	45	40	6	300	600	1000

PDT: Peak defining time, HDT: Hit defining time, HLT: Hit locking time.

## Data Availability

The datasets used and/or analyzed during the current study are available from the corresponding author on reasonable request.

## References

[1] Shah A.A., Ribakov Y. (2010). Effectiveness of nonlinear ultrasonic and acoustic emission evaluation of concrete with distributed damages. Mater. Des..

[2] Feiteira J. (2017). Monitoring crack movement in polymer-based self-healing concrete through digital image correlation, acoustic emission analysis and SEM in-situ loading. Mater. Des..

[3] Demis S., Papadakis V.G. (2019). Durability design process of reinforced concrete structures-Service life estimation, problems and perspectives. J. Build. Eng..

[4] Geng J., Sun Q., Zhang Y. (2017). Acoustic Emission Analysis of Deformation and Damage Processes. Constr. Build. Mater..

[5] Nair A., Cai C.S. (2010). Acoustic emission monitoring of bridges: Review and case studies. Eng. Struct..

[6] Carpinteri A., Lacidogna G., Pugno N. (2007). Structural damage diagnosis and life-time assessment by acoustic emission monitoring. Eng. Fract. Mech..

[7] Carpinteri A., Lacidogna G. (2006). Damage Monitoring of an Historical Masonry Building by the Acoustic Emission Technique. Mater. Des..

[8] Rehman S.K.U., Ibrahim Z., Memon S.A., Jameel M. (2016). Nondestructive test methods for concrete bridges: A review. Constr. Build. Mater..

[9] Singh N., Singh S.P. (2018). Evaluating the performance of self compacting concretes made with recycled coarse and fine aggregates using non destructive testing techniques. Constr. Build. Mater..

[10] Rasheed M.A., Prakash S.S., Raju G., Kawasaki Y. (2018). Fracture studies on synthetic fiber reinforced cellular concrete using acoustic emission technique. Constr. Build. Mater..

[11] Hu S., Lu J., Xiao F. (2013). Evaluation of concrete fracture procedure based on acoustic emission parameters. Constr. Build. Mater..

[12] Farnam Y., Geiker M.R., Bentz D., Weiss J. (2015). Acoustic emission waveform characterization of crack origin and mode in fractured and ASR damaged concrete. Cem. Concr. Compos..

[13] Suzuki T., Ohtsu M. (2004). Quantitative damage evaluation of structural concrete by a compression test based on AE rate process analysis. Constr. Build. Mater..

[14] Liu W., Guo Z., Niu S. (2020). Mechanical properties and damage evolution behavior of coal–fired slag concrete under uniaxial compression based on acoustic emission monitoring technology. J. Mater. Res. Technol.-JMRT.

[15] Abouhussien A.A., Hassan A.A.A. (2020). Hassan. Classification of damage in self-consolidating rubberized concrete using acoustic emission intensity analysis. Ultrasonics.

[16] Ma G., Wu C., Hwang H.-J., Li B. (2021). Crack monitoring and damage assessment of BFRP-jacketed concrete cylinders under compression load based on acoustic emission techniques. Constr. Build. Mater..

[17] Tayfur S., Alver N., Abdi S., Saatcı S., Ghiami A. (2018). Characterization of concrete matrix/steel fiber de-bonding in an SFRC beam: Principal component analysis and k-mean algorithm for clustering AE data. Eng. Fract. Mech..

[18] Behnia A., Chai H.K., GhasemiGol M., Sepehrinezhad A., Mousa A.A. (2019). Advanced damage detection technique by integration of unsupervised clustering into acoustic emission. Eng. Fract. Mech..

[19] Thirumalaiselvi A., Sasmal S. (2021). Pattern recognition enabled acoustic emission signatures for crack characterization during damage progression in large concrete structures. Appl. Acoust..

[20] Kravchuk R., Landis E.N. (2018). Acoustic emission-based classification of energy dissipation mechanisms during fracture of fiber-reinforced ultra-high-performance concrete. Constr. Build. Mater..

[21] Chen X., Xu Z. (2016). The ultrasonic P-wave velocity-stress relationship of rocks and its application. Bull. Eng. Geol. Environ..

[22] Sun Q., Zhu S. (2013). Wave velocity and stress/strain in rock brittle failure. Environ. Earth Sci..

[23] Zhang Z., Wang E., Dong C. (2016). Study on loaded rocks’ P-wave velocity anisotropy and quantitative relationship between the anisotropy index and the position of main failure. J. Appl. Geophys..

[24] Zhang Y., Abraham O., Grondin F., Loukili A., Tournat V., Le Duff A., Lascoup B., Durand O. (2012). Study of stress-induced velocity variation in concrete under direct tensile force and monitoring of the damage level by using thermally-compensated Coda Wave Interferometry. Ultrasonics.

[25] Zacchei E., Nogueira C.G. (2019). Chloride diffusion assessment in RC structures considering the stressstrain state effects and crack width influences. Constr. Build. Mater..

[26] Venkatalaxmi A., Padmavathi B.S., Amaranath T. (2004). A general solution of unsteady Stokes equations. Fluid Dyn. Res..

[27] Dong L.-J., Li X.B., Zhou Z.-L., Chen C.-H., Ma J. (2015). Three-dimensional analytical solution of acoustic emission source location for cuboid monitoring network without pre-measured wave velocity. Trans. Nonferrous Met. Soc. China.

[28] Mahboubi A., Ajorloo A. (2015). Experimental study of the mechanical behavior of plastic concrete in triaxial compression. Cem. Concr. Res..

[29] Jivkov A.P., Engelberg D.L., Stein R. (2013). Pore space and brittle damage evolution in concrete. Eng. Fract. Mech..

[30] Ran H., Guo Y., Feng G., Qi T., Du X. (2021). Creep properties and resistivity-ultrasonic-AE responses of cemented gangue backfill column under high-stress area. Int. J. Min. Sci. Technol..

[31] Havlásek P. (2021). Numerical modeling of axially compressed circular concrete columns. Eng. Struct..

[32] Song T.-Y., Xiang K. (2020). Performance of axially-loaded concrete-filled steel tubular circular columns using ultra-high strength concrete. Structures.

[33] Song Z., Konietzky H., Herbst M. (2019). Three-dimensional particle model based numerical simulation on multi-level compressive cyclic loading of concrete. Constr. Build. Mater..

[34] Sánchez-Aparicio L.J., Villarino A., García-Gago J., González-Aguilera D. (2016). Photogrammetric, Geometrical, and Numerical Strategies to Evaluate Initial and Current Conditions in Historical Constructions: A Test Case in the Church of San Lorenzo (Zamora, Spain). Remote Sens..

[35] Zhou K., Lei D., He J., Zhang P., Bai P., Zhu F. (2021). Real-time localization of micro-damage in concrete beams using DIC technology and wavelet packet analysis. Cem. Concr. Compos..

[36] Wang X., Jin Z., Liu J. (2021). Research on internal monitoring of reinforced concrete under accelerated corrosion, using XCT and DIC technology. Constr. Build. Mater..

[37] Dong W., Rong H., Wu Q., Li J. (2019). Investigations on the FPZ evolution of concrete after sustained loading by means of the DIC technique. Constr. Build. Mater..

[38] Okeil A., Matsumoto K., Nagai K. (2020). Investigation on local bond behavior in concrete and cement paste around a deformed bar by using DIC technique. Cem. Concr. Compos..

[39] Jin X., Tong J., Tian Y. (2018). Time-varying relative displacement field on the surface of concrete cover caused by reinforcement corrosion based on DIC measurement. Constr. Build. Mater..

[40] Babaeeian M., Mohammadimehr M. (2020). Investigation of the time elapsed effect on residual stress measurement in a composite plate by DIC method. Opt. Lasers Eng..

[41] Birch F. (1960). The velocity of compressional waves in rocks to 10 kilobars: 2. J. Geophys. Res.-Atmos..

[42] Castagna J.P., Batzle M.L., Eastwood R.L. (1985). Relationships between compressional-wave and shear-wave velocities in clastic silicate rocks. Geophysics.

[43] Shokouhi P., Zoëga A., Wiggenhauser H. (2010). Nondestructive Investigation of Stress-Induced Damage in Concrete. Adv. Civ. Eng..

[44] Shokouhi P., Zoëga A., Wiggenhauser H., Fischer G. (2012). Surface Wave Velocity-Stress Relationship in Uniaxially Loaded Concrete. ACI Mater. J..

[45] Lee J.-S., Yoon H.-K. (2015). Theoretical relationship between elastic wave velocity and electrical resistivity. J. Appl. Geophys..

[46] Clarke J., Adam L., van Wijk K., Sarout J. (2020). The influence of fluid type on elastic wave velocity and attenuation in volcanic rocks. J. Volcanol. Geotherm. Res..

[47] Meng K., Li Q. (2021). A homogenized damping model for the propagation of elastic wave in a porous solid. J. Sound Vibr..

